# optTMT: optimizing any experimental design to minimize false positives caused by TMT reporter ion interference

**DOI:** 10.1093/bioadv/vbaf243

**Published:** 2025-10-01

**Authors:** Marc-Antoine Gerault

**Affiliations:** Department of Oncology-Pathology, Karolinska Institutet, Stockholm 171 77, Sweden

## Abstract

**Summary:**

To enable multiplexing in large-scale proteomics experiment, TMT-plex isobaric tag has been the gold standard. However, tandem mass tag (TMT) reporter ion interference, also named cross-label isotopic impurity, can occur from manufacture level impurities and experimental error. Such interference increases the risk of false positive after differential analysis, even more so on high intensity peptide further leading to wrong conclusions. However, by planning the right experimental design beforehand these interferences can be minimized by solving an optimization problem. In this work, I present a *user-friendly* interface to allow the proteomics community to find any optimal TMT experimental designs.

**Availability and implementation:**

The Shiny application is available as an executable file at https://zenodo.org/records/14883262 for Windows users and at https://marc-antoinegerault.shinyapps.io/TMT_optimization/. Code and documentation are freely available on github at https://github.com/mgerault/optTMT. The package is written in R and a vignette shows its use in an R command line workflow.

## 1 Introduction

Study a whole proteome from cells or tissues is now more and more accessible to the scientific community. Using state-of-the-art mass spectrometer (MS) like the Orbitrap Astral MS coupled with data independent analysis enable to identify more than 8000 proteins in 8 min (ThermoFisher Scientific) making it possible in the future to perform proteomics analysis in the clinic. Proteomics experiments usually involve the comparison of different conditions in several replicates in order to analyze many characteristics of the proteome such as protein–protein interactions, protein subcellular localization or post-translational modifications (PTMs) (Larance and Lamond 2015). The MS data acquisition time of such experiments significantly increases as the number of samples rises, as well as risks of potential batch effects and noise induced by technical variations.

To overcome the issues brought by large-scale proteomics experiments, methods have been developed to allow the analyses of multiple samples in parallel. These methods involve the multiplexing of isotopically tagged peptides and the most widely used is the TMT-plex isobaric tags (Thompson *et al.* 2003). Multiplexed TMT enable to increase the sample throughput in proteomics experiment allowing labelling 10 to 18 or even up to 35 different samples in a single batch which significantly reduce the missing value rate arising from the stochastic sampling inherent to the MS data acquisition and mostly prevalent in data dependent acquisition (Webb-Robertson *et al.* 2015). Hence, within a single TMT batch, the number of missing values at the protein level is usually <2% with a high quantification precision (O'Connell *et al.* 2018).

Nevertheless, TMT-plex has been shown to have several drawbacks (Brenes *et al.* 2019). The one I will address here is the increase of the false positive rate due to the effect of the reporter ion interference, also named as cross-label isotopic impurity (Ow *et al.* 2009). This can arise from manufacture level impurities (Table 1, available as supplementary data at *Bioinformatics Advances* online) and experimental error. Where, for example, the reporter ion 127C contains 0.8% of the reporter ion 126% and 6.5% of the reporter ion 128C (Table 1, available as supplementary data at *Bioinformatics Advances* online). After data analysis, these impurities can lead to false positives and aberrant conclusions. For example, (Brenes *et al.* 2019) showed that by studying male and female samples in several TMT batches, they could quantify Y chromosome specific peptides in all TMT channels corresponding to the female samples and that the reporter ion interference can have significant effect on the quantification of high intensity peptides. Even though algorithms to correct this effect exist (Niu *et al.* 2017, Alves *et al.* 2024) and that proteomics quantification software like MaxQuant (Cox and Mann 2008) and Proteome Discoverer (ThermoFisher Scientific) provides such correction, the probability to detect and quantify peptides in conditions where they should not be, resulting in a greater false discovery rate, is still not minimized.

**Figure 1.**
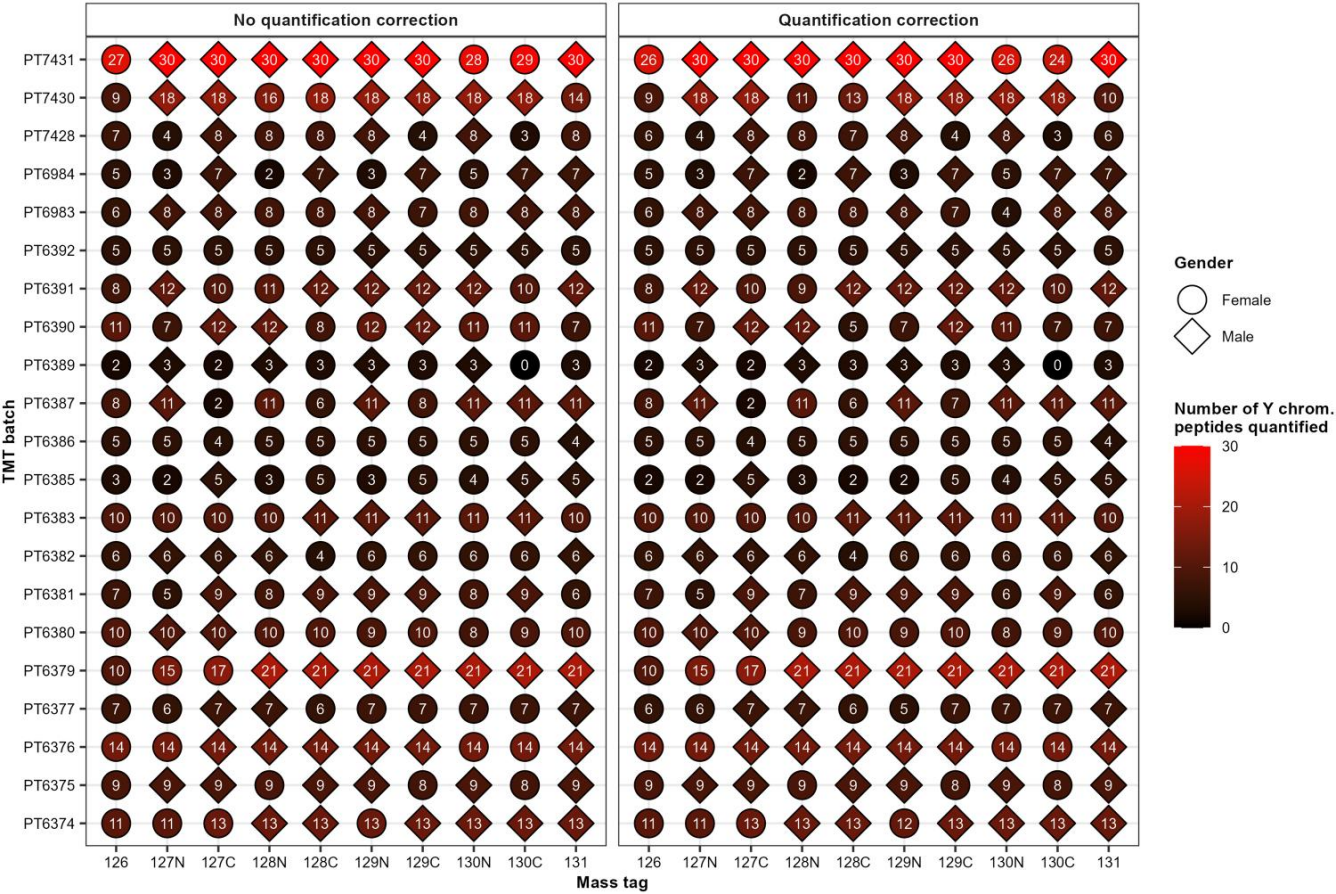
Number of quantified peptides with their corresponding master proteins matched to the set of the 11 protein coding genes uniquely located on the Y chromosome from the iPSC dataset (Kilpinen *et al.* 2017). The raw files from the 21 TMT batches from the iPSC dataset (Kilpinen *et al.* 2017) containing male and female samples were downloaded on PRIDE with the identifier PXD010557 and were then quantified using the Proteome Discoverer 3.2 (ThermoFisher Scientific) software. Quantification correction was applied using the product data sheet provided by ThermoFisher Scientific for the product number 90110, lot number UL298812. A peptide was considered quantified when its raw abundance was >0.

To demonstrate this problem, I reanalyzed the iPSC dataset produced by Kilpinen *et al.* (2017) and studied by Brenes *et al.* (2019). I performed peptide identification and quantification from the raw files of the 21 TMT batches containing male and female samples from the iPSC dataset provided by Brenes *et al.* (2019) using the Proteome Discoverer 3.2 (ThermoFisher Scientific) software. The workflow and FASTA file as well as the raw files used for this study and their corresponding results files from Proteome Discoverer are available at the ProteomeXchange Consortium via the PRIDE (Perez-Riverol *et al.* 2025) partner repository with the dataset identifier PXD063953. To reconfirm that TMT reporter ion interference has a significant impact on false positive detection, as already demonstrated by (Brenes *et al.* 2019), even with the use of quantification correction, I used the same method as depicted by (Brenes *et al.* 2019) where Y chromosome peptides were used as an internal control. The iPSC dataset was derived from 163 different donors including both male and female. Hence any Y chromosome specific peptides quantified in the TMT channels corresponding to female samples is a false positive and is due to reporter ion interference and/or coisolation interference. The set of 11 protein coding genes uniquely located on the Y chromosome used in this study was the same as the one used by (Brenes *et al.* 2019), i.e. CDY1B, CDY2B, DDX3Y, EIF1AY, KDM5D, NLGN4Y, PCDH11Y, RPS4Y1, TBL1Y, USP9Y, and UTY. For each of the 21 TMT batches with their 10 channels, the number of quantified peptides with its corresponding master proteins matched to the set of the 11 protein coding genes uniquely located on the Y chromosome was extracted (Fig. 1).

**Figure 2.**
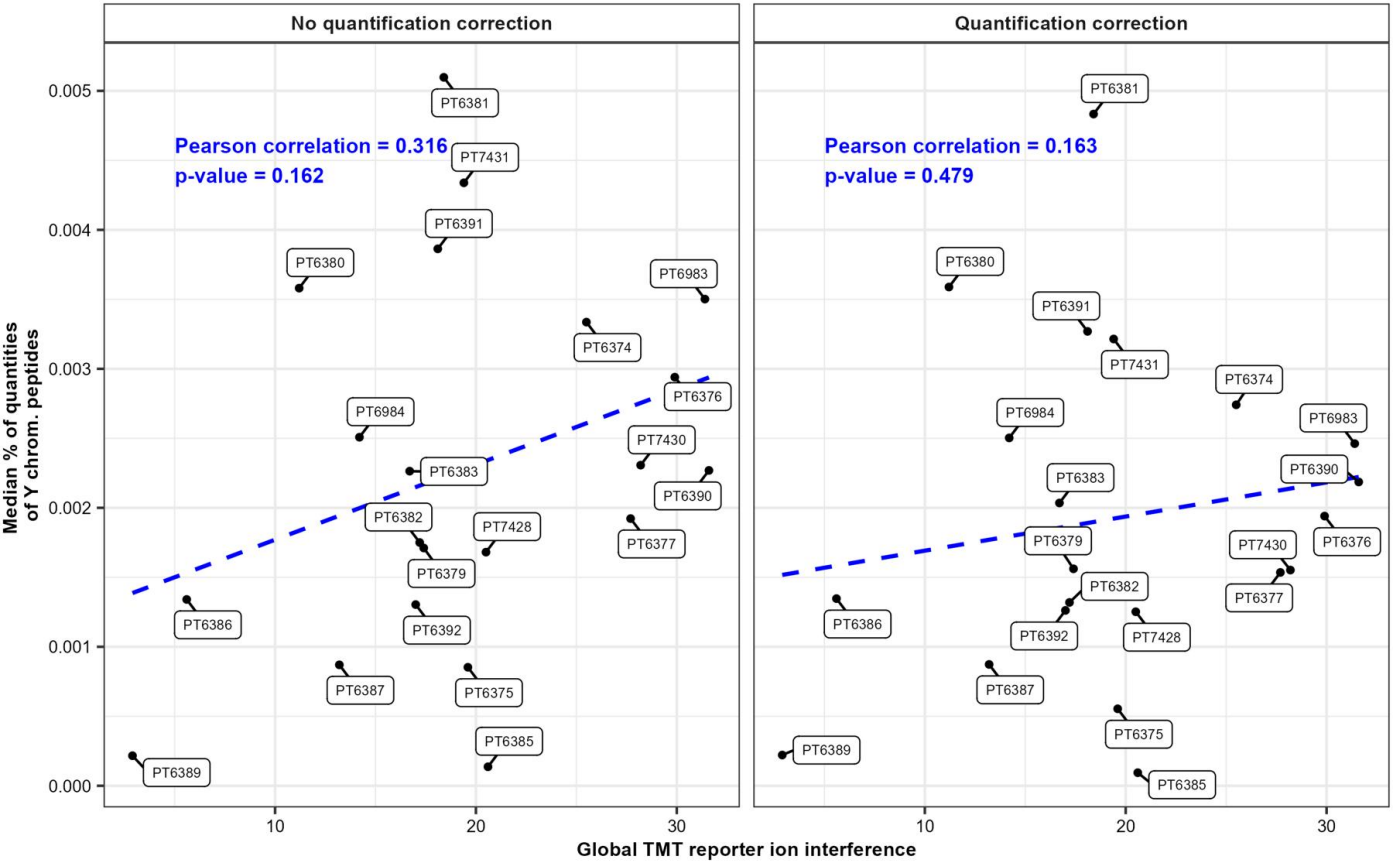
Correlation between the global TMT reporter ion interference of each TMT batch and their median of proportion of raw abundance of the peptides with their corresponding master proteins matched to the set of the 11 protein coding genes uniquely located on the Y chromosome from the iPSC dataset (Kilpinen *et al.* 2017). The raw files from the 21 TMT batches from the iPSC dataset (Kilpinen *et al.* 2017) containing male and female samples were downloaded on PRIDE with the identifier PXD010557 and were then quantified using the Proteome Discoverer 3.2 (ThermoFisher Scientific) software. Quantification correction was applied using the product data sheet provided by ThermoFisher Scientific for the product number 90110, lot number UL298812. The global TMT reporter ion interference was obtained by summing the impurities of TMT reporter ion corresponding to a male sample in each channel corresponding to a female sample.

Figure 1 shows that the number of quantified Y chromosome peptides in female samples stays equivalent for the majority of the samples after using quantification correction. To further investigate the impact of the experimental design and the quantification correction, I extracted the median of the proportion of raw abundance of the quantified Y chromosome peptides in female samples in each of the TMT batches. Figure 2 show the correlation between the amount of quantified false positive and the total reporter ion interference generated by the experimental design, which I will name global interference, for each TMT batch of the iPSC dataset. The global interference was obtained by summing the impurity level given by ThermoFisher Scientific (Table 1, available as supplementary data at *Bioinformatics Advances* online) of each channel occurring between a male and female sample. Although the quantification correction did not reduce the number of quantified Y chromosome peptides in male samples (Fig. 1), it did reduce their abundance in most TMT batches (Fig. 2). Coisolation interference is also at play here reducing the significance of the correlation between the global interference of experimental design and the amount of false positive but we do observe a positive correlation, even after quantification correction (Fig. 2).

Hence, to reduce as much as possible false positives due to reporter ion interference, one can select an appropriate experimental design beforehand that will minimize the global interference. Indeed, most of the channel leakage is occurring between the corresponding +1 or −1 isotope N or C of the channel. For example, the channel 130 N contains impurities of the reporter ions 129 N and 131 N whereas the channel 130C contains impurities of only the reporter ion 129C in a TMT10-plex. By using the impurities data from the manufacturer as presented in Table 1, available as supplementary data at *Bioinformatics Advances* online, one can find the optimal experimental design that will minimize the rate of false positive. Additionally, if a correction algorithm is used for the protein quantification, it will now minimize the noise between replicates, allowing better quantification and lower coefficient of variations. (Brenes *et al.* 2019) did propose some optimal designs for simple experiments using TMT10 or TMT11 but generalize them for any experimental set up is a harder task. Moreover, with now the possibility to use TMT16 or even 35 plex, finding the optimal experimental design is far more complex, even more so depending on the number of different samples. For a set X of size Ncontaining k unique conditions each with its associated number of replicates $n_{1},\;n_{2},\;\ldots ,n_{k}$, the number of possible permutations, i.e. the number of potential experimental designs D is given by $D=\frac{N!}{\prod_{i=1}^{k}n_{i}!}$. Thus for a TMT10-plex set with 2 conditions with 5 replicates each, the number of possible designs is only 252. On the other hand, for a TMT16-plex set with 5 conditions in triplicates and a reference channel, the number of possible designs rise to 2 690 688 000. Whereas the computation of all possible designs is impossible in this case, finding an, or the, optimal design with such (or any) parameters is feasible by solving an optimization problem.

## 2 The optimization problem

Let there be, for a given experiment, N the number of channels, k the number of conditions, $n_{1},\;n_{2},\;\ldots ,n_{k}$ their associated number of replicates and $\alpha_{j}$, $\beta_{j}$ the reporter ion interference value to the corresponding +1 or −1 isotope of the channel j. Let’s also call $x_{i,j}\in \{0,1\}$ the variable reflecting the fact that the condition i is assigned to the channel j. From here, we can deduce the following properties and constraints of the problem.

First, we have the property $P_{1}$ stating that $\beta_{1}=\beta_{2}=NA$ since there is no corresponding −1 isotope for the two first channels and that $\alpha_{N-1}=\alpha_{N}=NA$ since there is no corresponding +1 isotope for the two last channels. Then, since we want to minimize the rate of false positive we need to consider interferences only when it’s occurring between different conditions. Thus, we have the property $P_{2}$ that if $x_{i,j}=x_{i,j+2}=1$ then $\alpha_{j-2}=\beta_{j+2}=0$ for the channel j and $\alpha_{j}=\beta_{j+4}=0$ for the channel j+2, this ∀ i∈{1, …, k} and ∀ j∈{1, …, N}. Next, we need to satisfy that a channel can only be assigned to one unique sample and that a condition i needs to have $n_{i}$ replicates and this ∀ i∈{1, …, k}. Therefore we can write the first constraint $C_{1}$ as $C_{1}:\;\sum_{i=1}^{k}x_{i,j}\leq 1\;\forall \;j\in \{1,\;\ldots ,\;N\}$ and the second constraint $C_{2}$ as $C_{2}:\;\sum_{j=1}^{N}x_{i,j}=n_{i}\;\forall \;i\in \{1,\;\ldots ,\;k\}$. Finally, we need to find the configuration X of $x_{i,j}$ that minimize the global reporter ion interference satisfying the constraints and given the properties defined above. The optimization can be written as follow: $\underset{X}{\mathrm{argmin}}(\sum_{i=1,j=1}^{k,N}(\alpha_{j}+\beta_{j}).x_{i,j})$satisfying the constraints $C_{1}$ and $C_{2}$ and given the properties $P_{1}$ and $P_{2}$.

As discussed previously, compute all possible configurations to find the configuration X solution of the problem is impossible. However, to find a local optimum, the neighbours of X can easily be obtained by computing all unique configurations X′ where two positions of X have been swapped. The number of possible neighbours Sn of X is given by $|Sn|=\frac{N^{2}-N}{2}-\sum_{i=1}^{k}\frac{n_{i}^{2}-n_{i}}{2}$. Since $1\leq |Sn|\leq \frac{N^{2}-N}{2}$, computing the global interference of all the neighbours of X is more than manageable considering TMT can have at the moment at most 35 different channels making 1≤|Sn|≤595.

Ultimately the problem can be solved using the following algorithm: given a configuration X, i.e. an experimental design, obtain all its possible neighbours Sn and compute their respective global interference. If a configuration X′ has a lower global interference, X become X′. If not, X can be randomly shuffled to restart the search for a better configuration. The procedure can be repeated until no better configuration is found or after a certain number of iterations.

## 3 The Shiny application

To allow any users finding the best experimental design for their proteomics experiment using TMT, I created a Shiny application named optTMT, based on the R language where, according to the variables of their experiment, user can know what experimental design is preferable. The source code of the optimization algorithm described in the previous section used by optTMT is available at https://github.com/mgerault/optTMT in the function *tmt_optimal*. Its time performance and memory allocation depending on the TMT set, the number of conditions and replicates is shown in Fig. 1, available as supplementary data at *Bioinformatics Advances* online. Figure 1, available as supplementary data at *Bioinformatics Advances* online shows that the computation time of *tmt_optimal* mostly depends on the size of the TMT selected where it ranges from 0 to 10 s for TMT10 to TMT18 and 0-3 min for TMT32 and TMT35 depending on the number of conditions and replicates.

**Figure 3.**
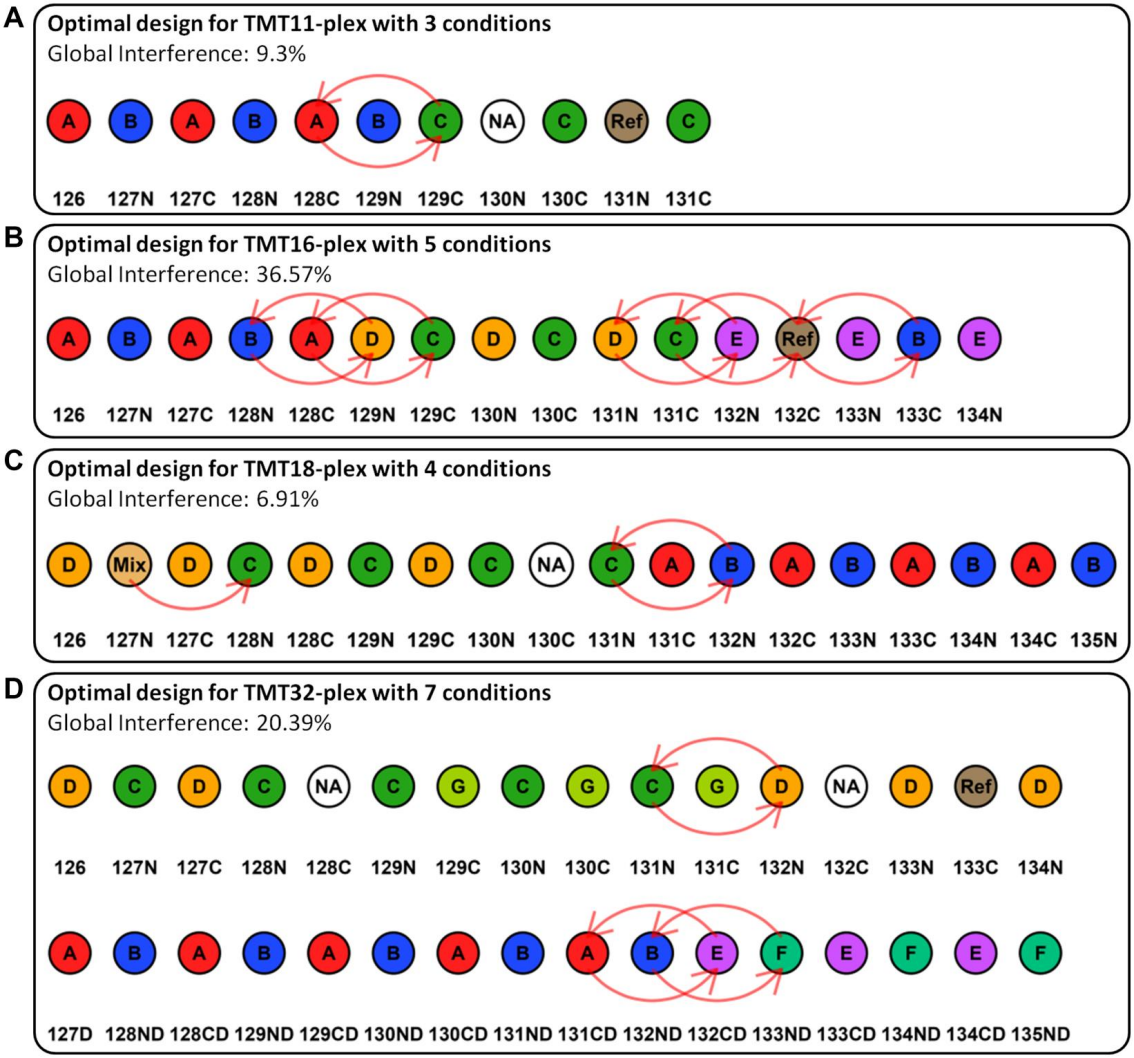
Examples of optimal experimental design to minimize the TMT reporter ion interference obtained using optTMT. (A) Optimal design for TMT11-plex with three conditions in triplicate, a reference channel and an empty channel. (B) Optimal design for TMT16-plex with five conditions in triplicate and a reference channel. (C) Optimal design for TMT18-plex with four conditions in quadruplicate, a reference channel and an empty channel. (D) Optimal design for TMT32-plex with four conditions in quintuplicate, three condition in triplicate, a reference channel and an empty channel.

TMT reporter ion interference data is already available in the application but the user can also choose to upload its own via an xlsx, csv or txt tab delimited file or even upload directly the ‘Product Data Sheet’ in pdf from ThermoFisher Scientific (Table 1, available as supplementary data at *Bioinformatics Advances* online). Furthermore, the user can eventually select the number of carrier channel. If a channel is empty, then it does not produce any interference. A carrier channel, on the other hand, is usually a channel containing the same sample in multiple TMT batches to increase the probability to identify the same proteins between different TMT batches but is not used for quantification normalization, as in (Dai *et al.* 2018). Hence, the only interference taken into account for this type of channel is the one it induces to other channels. Such channels are labelled ‘Mix’ by default in optTMT (Fig. 3). Although if a reference channel is needed for TMT batch correction and normalization, the interference it induces to other channels and the one it receives from others needs to be taken into account, i.e. it should be considered as a unique different condition (Fig. 3).

Once the parameters have been selected, the optimal design can be computed, plotted and downloaded. For example, when selecting the same parameters corresponding to the PT6389 experiment shown in Fig. 1 or to the optimal designs proposed by the Fig. 6 of Brenes *et al.* (2019), the same or even better optimal designs are returned by optTMT (Fig. 2, available as supplementary data at *Bioinformatics Advances* online). Additionally, when downloading the design, all possible equivalent designs resulting in the same global interference will be returned. Indeed TMT batches, if several are needed, should be randomized to accurately estimate and correct batch effect, even more so in a single cell proteomics experiment (Vanderaa and Gatto 2021, Gatto *et al.* 2023). However, if the user has too many conditions to fit one TMT experiment and has to use several batches, the user can also select more than one batch. optTMT will then assign the samples to each batch so that they have the smallest number of different conditions possible and finally optimize each batch individually. Typical optimal experimental designs obtained thanks to optTMT are shown in Fig. 3.

The Shiny application is directly usable as a web application at https://marc-antoinegerault.shinyapps.io/TMT_optimization/. However, since it is hosted on the free server shinyapps.io, the operating time is limited. If the user plans to run several test or want to use optTMT regularly, the application is also available as a standalone software at https://zenodo.org/records/14883262 and its source code can be found as an R package as well at https://github.com/mgerault/optTMT.

## 4 Conclusion

As demonstrated by (Brenes *et al.* 2019) and the work presented here, TMT reporter ion interference can have a significant impact on the interpretation of the results of a proteomics experiment, even when TMT quantification correction is applied. However, the increase of false positives due to reporter ion interference can be minimized beforehand by selecting an optimal experimental design. But finding such an experimental design can be an arduous task. To answer this matter I provide optTMT, a tool to easily obtain any optimal TMT experimental design depending on the experiment parameters of any users. As TMT-plex is still widely used in the proteomics community, this *user-friendly* interface will allow to easily plan the best experimental design and ultimately lowering the false positive rate in proteomics experiment using TMT multiplexing.

## Supplementary Material

vbaf243_Supplementary_Data

## Data Availability

The Shiny application is available as an executable file at https://zenodo.org/records/14883262 for Windows users and at https://marc-antoinegerault.shinyapps.io/TMT_optimization/. Code and documentation are freely available on github at https://github.com/mgerault/optTMT. The package is written in R and a vignette shows its use in an R command line workflow. The workflow and FASTA file as well as the raw files used for this study and their corresponding results files from Proteome Discoverer are available at the ProteomeXchange Consortium via the PRIDE (Perez-Riverol *et al.* 2025) partner repository with the dataset identifier PXD063953. R code used to analyze Proteome Discoverer output data and generate figures can be obtained upon request to the corresponding author.
