# Supplementary material for: optTMT: optimizing any experimental design to minimize false positives caused by TMT reporter ion interference
Source: Bioinform Adv. 2025 Oct 1;6(1):vbaf243. doi: 10.1093/bioadv/vbaf243 (PMC12975717; doi:10.1093/bioadv/vbaf243)
Supplement: vbaf243_Supplementary_Data [file vbaf243_supplementary_data.zip › Supplementary Materials - Gerault - Bioinformatics Advances - optTMT optimize TMT reporter ion interference.pdf]

Supplementary Table 1. Typical product data sheet from ThermoFisher Scientific giving the reporter ion interference for TMT10plex label reagent set. The data shown here correspond to the product number 90110, lot number UL298812.

Mass Tag	Reporter Ion	-2	-1	+1	+2
**TMT^{10}-126**	126.127726	NA	NA	7.4% (127C)	0.0% (128C)
**TMT^{10}-127N**	127.124761	NA	NA	6.2% (128N)	0.0% (129N)
**TMT^{10}-127C**	127.131081	NA	0.8% (126)	6.5% (128C)	0.0% (129C)
**TMT^{10}-128N**	128.128116	NA	0.4% (127N)	6.6% (129N)	0.0% (130N)
**TMT^{10}-128C**	128.134436	0.0% (126)	1.5% (127C)	5.8% (129C)	0.0% (130C)
**TMT^{10}-129N**	129.131471	0.0% (127N)	1.6% (128N)	5.4% (130N)	0.0% (131)
**TMT^{10}-129C**	129.137790	0.0% (127C)	2.7% (128C)	4.7% (130C)	NA
**TMT^{10}-130N**	130.134825	0.0% (128N)	2.4% (129N)	4.4% (131)	NA
**TMT^{10}-130C**	130.141145	0.0% (128C)	3.3% (129C)	NA	NA
**TMT^{10}-131**	131.138180	0.8% (129N)	2.9% (130N)	NA	NA

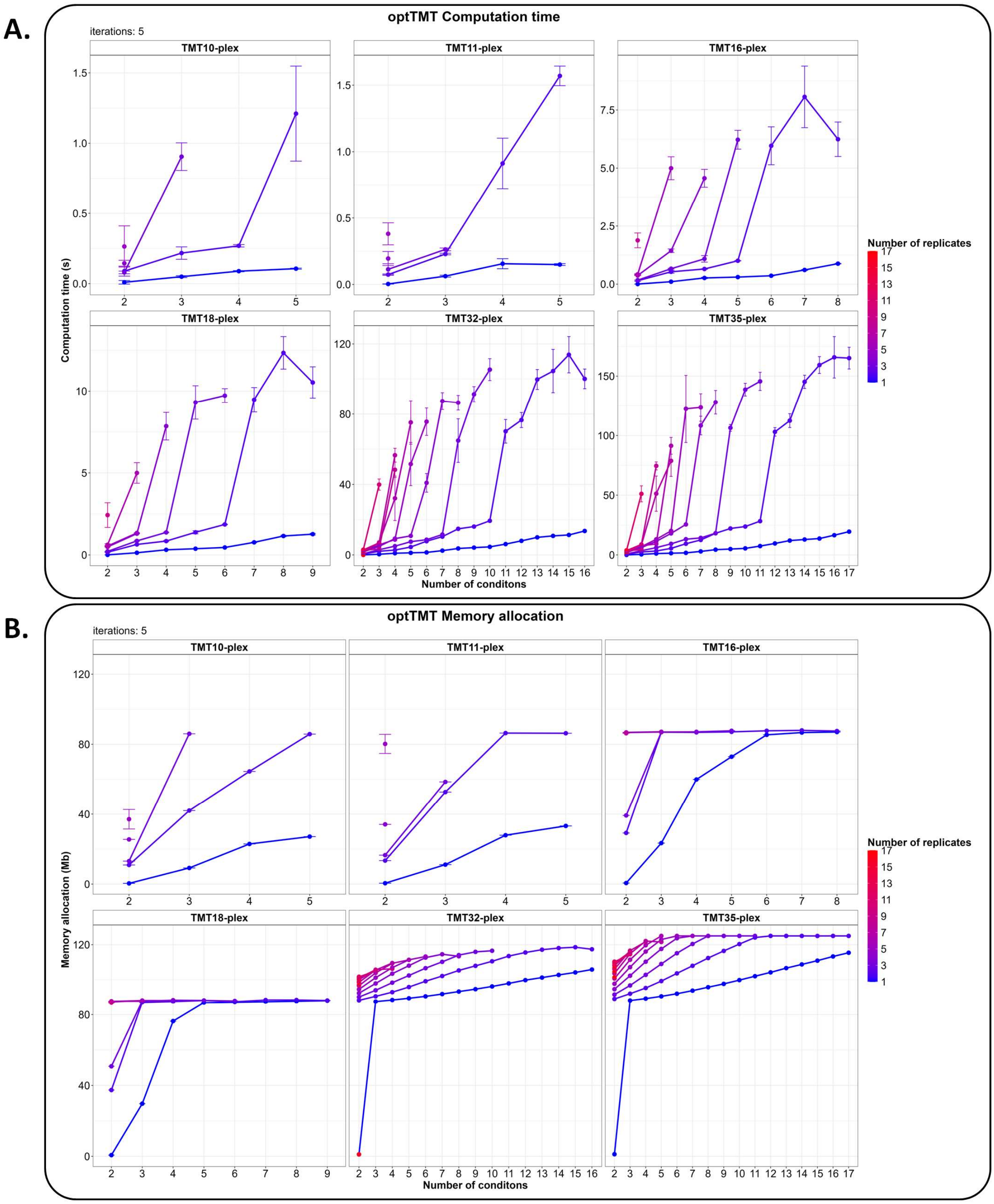

Supplementary Figure 1. Time performance and memory allocation of the function *tmt_optimal* from optTMT depending on the TMT set, the number of conditions and replicates selected. (A) Time performance in seconds of the *tmt_optimal* function from optTMT depending on the selected parameters. 5 iterations were run to obtain the error bars. **(B)** Memory allocation in Mb of the *tmt_optimal* function from optTMT depending on the selected parameters. 5 iterations were run to obtain the error bars.

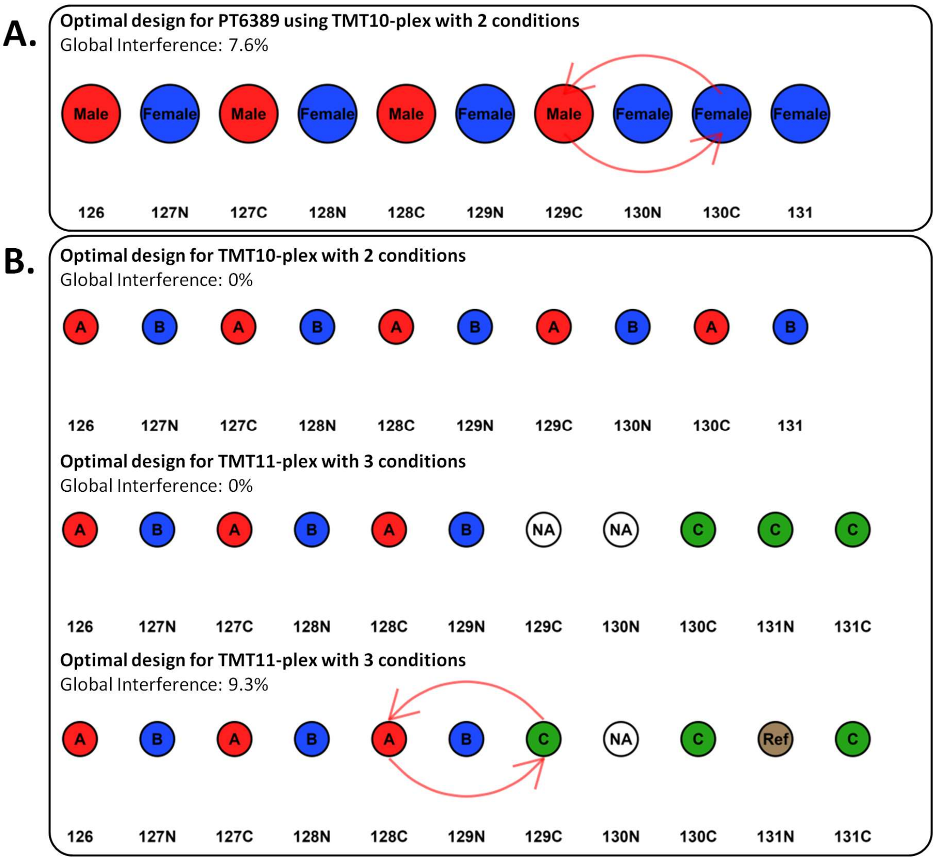

Supplementary Figure 2. Optimal experimental design obtained using optTMT corresponding to the designs of the PT6389 experiment and the ones proposed in figure 6 of (Brenes et al, 2019). (A) Optimal design for the PT6389 experiment from (Kilpinen et al. 2017) using TMT10-plex with 4 male samples and 6 female samples. **(B)** Optimal designs corresponding to the 3 designs proposed by (Brenes et al., 2019) in figure 6 to minimize reporter interference with either 2 or 3 conditions and eventually a reference channel using either TMT10-plex or TMT11-plex.
